# Bilateral cranioorbital foramina (Hyrtl foramina): crucial anatomical findings in the management of giant olfactory groove meningioma - a case report and literature review

**DOI:** 10.1093/jscr/rjae476

**Published:** 2024-08-24

**Authors:** Mohammad Khalil Al-Barbarawi, Amr Badary, Wahab Moustafa, Oday Atallah, Karsten Stock, Piotr Czapiewski, Hans-Christof Renner

**Affiliations:** Department of Neurosurgery, Dessau Medical Center, Auenweg 38,06847 Dessau-Roßlau, Brandenburg University, Dessau-Roßlau, Germany; Department of Neurosurgery, Dessau Medical Center, Auenweg 38,06847 Dessau-Roßlau, Brandenburg University, Dessau-Roßlau, Germany; Department of Neurosurgery, Dessau Medical Center, Auenweg 38,06847 Dessau-Roßlau, Brandenburg University, Dessau-Roßlau, Germany; Department of Neurosurgery, Hannover Medical School, Carl-Neuberg-Straße 1, 30625 Hannover, Germany; Department of Radiology, Dessau Medical Center, Auenweg 38,06847 Dessau-Roßlau, Brandenburg University, Dessau-Roßlau, Germany; Department of Pathology, Dessau Medical Center, Auenweg 38,06847 Dessau-Roßlau, Brandenburg University, Dessau-Roßlau, Germany; Department of Neurosurgery, Dessau Medical Center, Auenweg 38,06847 Dessau-Roßlau, Brandenburg University, Dessau-Roßlau, Germany

**Keywords:** bilateral cranioorbital foramina, Hyrtl foramina, giant olfactory groove meningioma, anatomical findings, neurosurgery

## Abstract

Presence of bilateral cranio-orbital foramina, AKA Hyrtl foramina is rare yet existing. They carry the risks of retinal artery emboli due to reflux embolization for the neurovascular interventionalists, navigating complexities in olfactory groove meningioma management. A 59-year-old woman with progressive frontal lobe syndrome presented a large olfactory groove meningioma primarily supplied by bilateral sphenopalatine arteries together with bilateral anterior cerebral arteries, necessitating risky preoperative embolization and meticulous resection. This case underscores the intricate nature of vascular supply in frontal skull base tumors, emphasizing the need for multidisciplinary approaches, thorough preoperative planning, and detective research to optimize treatment outcomes.

## Introduction

The complexity of arterial blood supply of fronto-basal space occupying lesion (SOL) variations poses challenges for neurosurgeons, neurovascular interventionalists, and neuroradiologists. Recent investigations reveal anastomoses between external carotid artery (ECA) and internal carotid artery (ICA) via bilateral sphenopalatine arteries with bilateral anterior cerebral arteries. Notably, the anastomosis prevalence between the orbital branch of the middle meningeal artery and the lacrimal artery through the cranio-orbital foramina (COF) ranges from 28% to 82.9%, with meta-analysis showing unilateral prevalence in 74% and bilateral in 26% [1].

Resecting olfactory groove meningiomas (OGMs) requires precision due to nearby vital structures and potential vascular variations to avoid complications. Comprising 10% of intracranial meningiomas, OGMs originate in the olfactory fossaa complex anatomical region resembling a ‘canyon, ‘with the frontal sinus as its main entrance.

This knowledge is crucial for ophthalmic and neurosurgeons tackling rare skull base procedures and tumor resections. The study examines COF prevalence and addresses complexities of OGMs, where vascular intricacies add complexity. Understanding rare arterial variants within OGMs requires nuanced comprehension, blending orbital anatomy with vascular challenges, including preoperative embolization.

## Case presentation

A 59-year-old woman was referred to our hospital’s emergency department due to progressive frontal lobe syndrome persisting for 4 months, characterized by memory disturbance, headache, and confusion, with no significant medical history. Clinical examination revealed hyposmia and diplopia attributed to minimal papilledema, hindering evaluation of the visual field due to the patient’s confusion.

**Figure 1:**
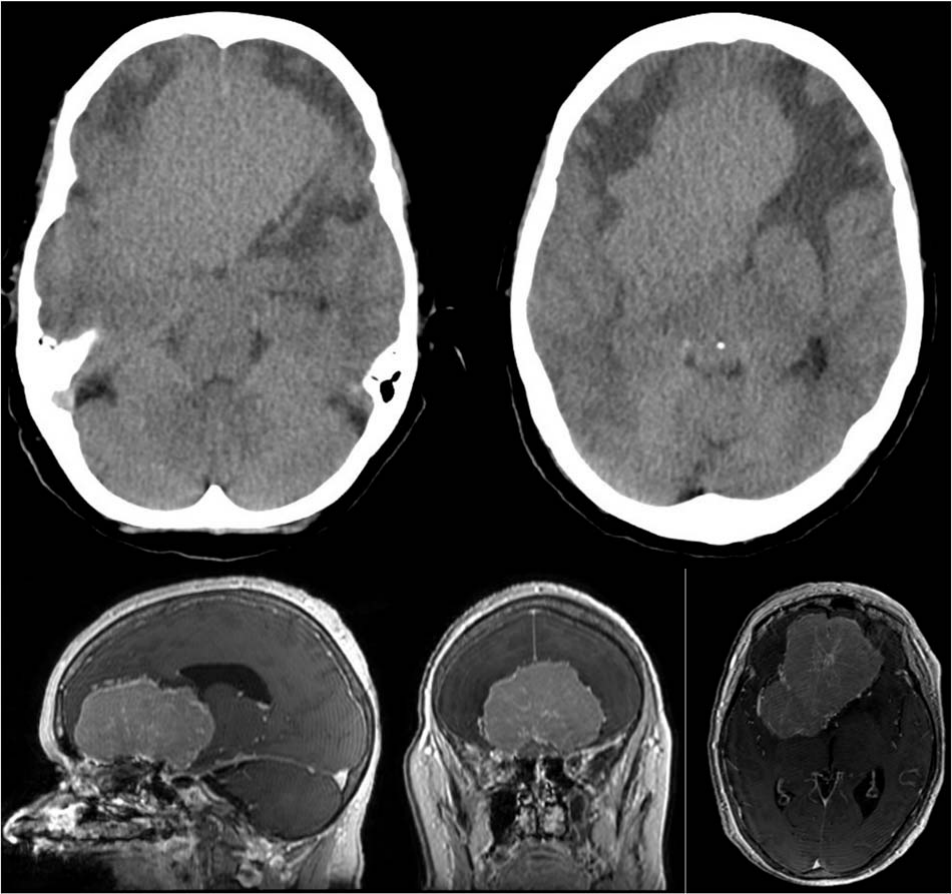
Showing the CT imaging with a hypodense fronto-basal lesion with finger-shaped perifocal edema and the T1-weighted MRI image with homogenous contrast-enhancing frontobasal lesion demonstrating mild compression on both lateral ventricles and posterior displacement of the right middle cerebral artery (MCA) without evidence of hydrocephalus.

**Figure 2:**
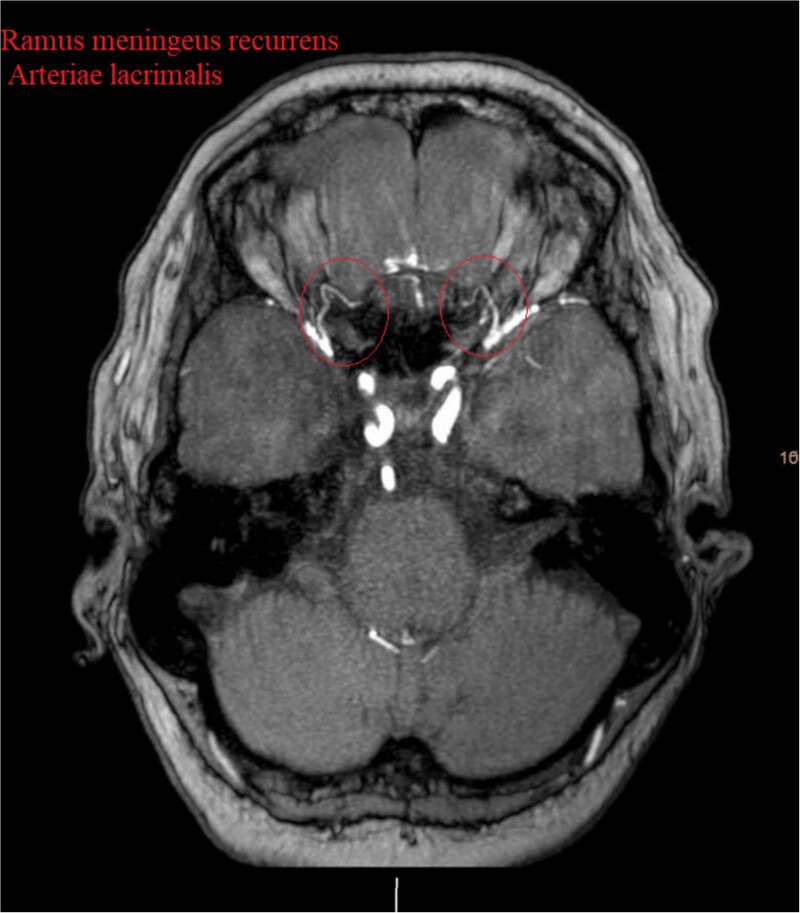
Show the MRA with the bilateral anastomotic branch of the lacrimal artery with the middle meningeal artery.

Brain CT imaging unveiled a hypodense fronto-basal lesion measuring 9–10 cm with finger-shaped perifocal edema (Fig. 1). contrast-enhanced cMRI provided more precise dimensions of the lesion (52 x 41 x 82 mm), demonstrating mild compression on both lateral ventricles and posterior displacement of the right middle cerebral artery and both anterior cerebral arteries without signs of hydrocephalus or perfusion disturbances (Fig. 1) and the bilateral COF anastomoses (Fig. 2) .

**Figure 3:**
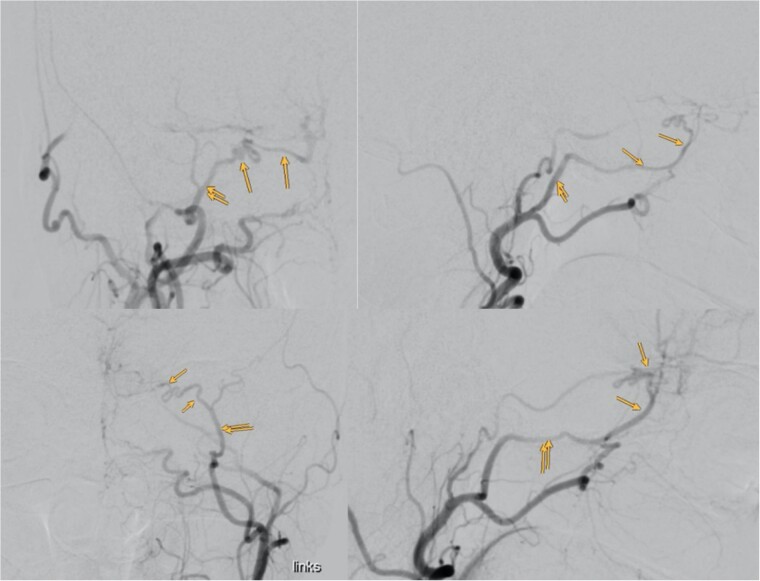
Showing the digital subtraction angiography of the left external carotid artery in four perspectives: posteroanterior view (upper left), lateral view (upper right), posteroanterior view (lower left), and lateral view (lower right). Additionally, the figure shows the middle meningeal artery and the anastomosis, which are marked.

Despite normal blood hormonal status except for low cortisone levels, the patient’s confusion improved upon initiation of physiological and pharmacological cortisone replacement therapy. Digital subtraction angiography revealed that the meningioma was predominantly supplied by bilateral sphenopalatine arteries and bilateral anterior cerebral arteries, with anastomosis through the COF (Fig. 3).

Preoperative embolization of the maxillary and sphenopalatine artery branches bilaterally via ECAs was performed to reduce blood loss risks. However, to avoid the risk of Gelfoam reflux emboli leading to blindness, only the nearest terminal branches to the meningioma were partially embolized.

Surgical excision was accomplished via bifrontal trepanation across the sinuses, with a total blood loss of 500 ml monitored intraoperatively. Throughout the operation, sensory and motor evoked potentials remained stable, except for a slight reduction in the amplitude of the motor evoked potentials of the left tibialis anterior.

**Figure 4:**
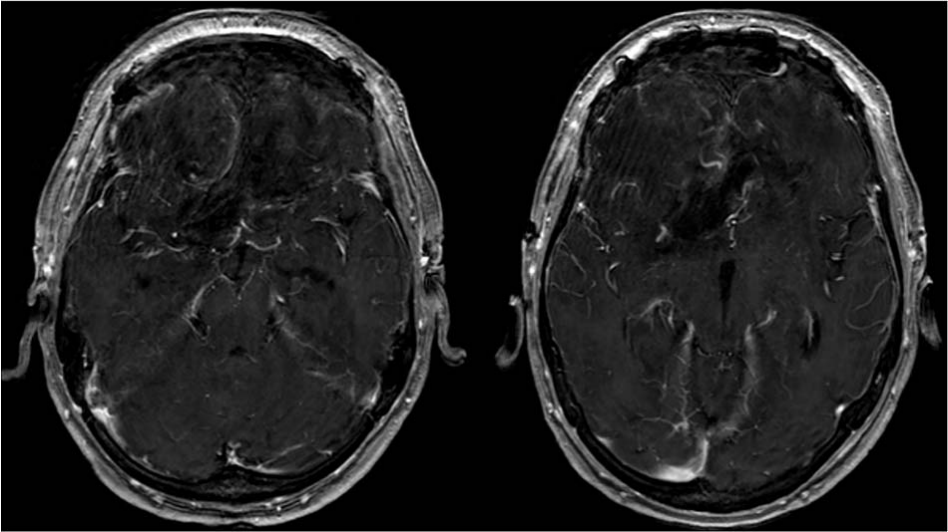
Showing the postoperative T1-weighted MRI with no residual tumor tissue.

**Figure 5:**
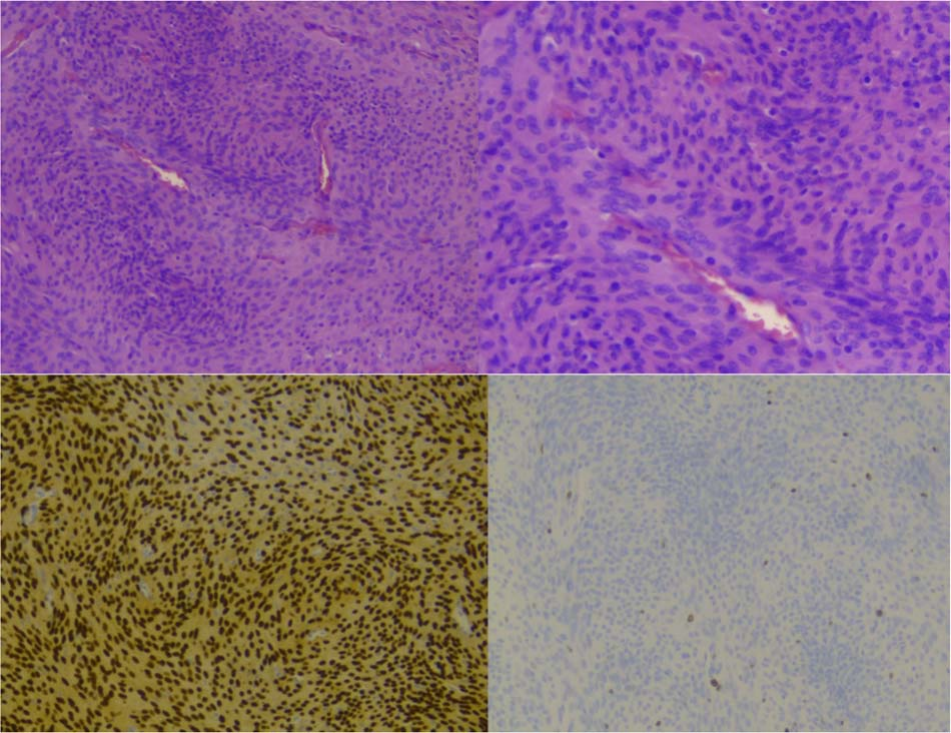
Showing staining with hematoxylin and eosin, low power lens (upper left), high power lens (upper right) and moleculopathological analysis with ki67 (lower left), progesteron receptor (lower right).

Postoperative cMRI showed typical postoperative changes with no residual tumor tissue (Fig. 4), prompting initiation of extubation and weaning. Histopathological analysis confirmed a meningothelial meningioma (WHO grade I) (Fig. 5) with no indication for further treatment. Discharge occurred after 12 days, with subsequent 6-month follow-up revealing no recurrence and improved cognitive function.

## Discussion

Meningiomas WHO grade I originate from the dural lining of the anterior cranial fossa, including the ethmoidal cribriform plate, fronto-sphenoidal suture, and planum sphenoidale. Common initial symptoms encompass headaches, visual disturbances, personality changes, and anosmia with variable duration [2].

Preoperative angiography is vital in determining the location and extent of displacement of the anterior cerebral circulation. It aids neurosurgeons in discerning the tumor’s vascular architecture, often focusing on the A2 segment, which may experience posterior or superior displacement. Preoperative embolization techniques have enhanced surgical approaches, reducing intraoperative blood loss and facilitating lesion resection [3].

Our case’s angiography revealed the meningioma’s vascular supply from bilateral sphenopalatine arteries and bilateral anterior cerebral arteries, with anastomosis through COF (Fig. 3). Catheterizing the ECA with such anastomosis poses challenges, especially near the ophthalmic artery. Caution during procedures, proper catheter positioning, and gentle injection techniques are crucial for safe embolization [4].

In our case, a notable feature was the circular anastomosis among arterial branches, reminiscent of the unique Hyrtl anastomosis [5]. This configuration resembled the meningeolacrimal variant of the Ramus lacrimalis originating from the middle meningeal artery, traversing the Hyrtl canal (HC) [1]. The HC, positioned variably lateral to the superior orbital fissure, is linked to the persistence of an embryonic canal for the supraorbital division of the stapedial artery, forming a canal for bilateral anastomosis between the orbital branch of the middle meningeal artery and the lacrimal branch of the ophthalmic artery in 26% of cases [1]. This finding offers a unique perspective on the vascular considerations associated with our case’s meningioma blood supply.

Chee *et al.’s* retrospective review of nine meningioma patients concluded that mental deterioration recovery could be expected post-tumor removal. Olfactory morbidity concerns in skull base surgery, with ~10% experiencing anosmia a year post-surgery, necessitate individual tailoring to minimize such morbidity [6]. Resection of OGM may improve visual impairment, with the duration of preoperative visual symptoms impacting postoperative recovery [7].

The personalized approaches in OGM management, refining techniques, and minimizing complications for optimal patient outcomes are always important. Advancements in personalized care and surgical precision promise improved results for patients with complex conditions.

## Conclusion

This case underscores the significance of thorough preoperative planning, meticulous surgical technique, and vigilant postoperative monitoring for achieving positive outcomes in patients with skull-base tumors with rare blood supply variants such as bilateral COF. A multidisciplinary approach is crucial for successful treatment.
